# Ascorbic Acid Interaction With Analgesic Effect of Morphine and Tramadol in Mice

**DOI:** 10.5812/aapm.19529

**Published:** 2014-06-22

**Authors:** Fatemeh Zeraati, Malihe Araghchian, Mohammad Hadi Farjoo

**Affiliations:** 1 Department of Pharmacology, Hamedan University of Medical Sciences, Hamedan, Iran

**Keywords:** Ascorbic Acid, Tramadol, Morphine

## Abstract

**Background:**

Combining different analgesic drugs for improvement of drug efficacy is a recommended strategy intended to achieve the optimal therapeutic effects.

**Objectives:**

The purpose of the present study was to assess the nature of the interaction between ascorbic acid and two analgesic drugs, morphine and tramadol.

**Materials and Methods:**

The analgesic activity was assessed by the acetic acid writhing test in male Naval Medical Research Institute (NMRI) mice. The results were obtained using four to six animals in each group. All the drugs were injected intraperitoneally. The effective doses (ED) that produced 20%, 50%, and 65% antinociception (ED_20_, ED_50_ and ED_65_) were calculated from the dose-response curve of each drug alone as well as co-administration of ascorbic acid and tramadol or morphine. The interaction index was calculated as experimental ED/theoretical ED. For each drug combination, ED_50_, ED_20_ and ED_65_ were determined by linear regression analysis of the dose-response curve, and they were compared to theoretical ED_50_, ED_20_ and ED_65_ using t-test.

**Results:**

The antinociceptive effects of all drugs were dose-dependent (ED_50_was 206.1 mg/kg for ascorbic acid, 8.33 mg/kg for tramadol, and 0.79 mg/kg for morphine). The interaction index demonstrated additive effects at ED_50_ and ED_65_ for co-administration of ascorbic acid and tramadol or morphine. However, at ED_20_, combination of ascorbic acid and tramadol or morphine showed synergic effects. The interaction index values of the combinations demonstrated the potency ratio of ascorbic acid/morphine to be lower than ascorbic acid/tramadol.

**Conclusions:**

This study demonstrated the results of interactions between ascorbic acid and tramadol or morphine. The results showed that the interaction effects on antinociception may be synergistic or additive, depending on the level of effect.

## 1. Background

Combining different analgesic drugs for improving the drug efficacy is a recommended strategy, intended to achieve optimal therapeutic effects. These strategies that are commonly used in almost all clinical settings are aimed to facilitate compliance in patients, simplify drug prescription, and decrease the drugs side effects, concomitant with increasing their efficacy (1, 2). In this context, concurrent administration of antinociceptive drugs can lead to achieving synergistic effects (3).

Several investigations have been carried out on physiological benefits of ascorbic acid and its applications as a therapeutic agent in various disease states. Furthermore, the beneficial effects of ascorbic acid as a cost-effective, convenient, and safe supplement for detoxifying narcotic drugs had been identified for many years (4). Regarding the antinociceptive effects of ascorbic acid, it was earlier reported that a single dose of ascorbic acid intraperitoneally can produce antinociception by interaction with ionotropic glutamate receptors (5). Moreover, a recent study demonstrated that plasma concentrations of ascorbic acid were lower in patients with neuralgia than in healthy volunteers and ascorbic acid treatment decreased the spontaneous pain in those patients (6, 7). It has been recently suggested that administration of ascorbic acid in combination with vitamin E can exert synergistic antinociceptive effects (8, 9) and it was reported that vitamin C exerted its antinociceptive effects primarily as a result of its antioxidant properties. It is clear that vitamin C has a major role in mitigating pain in a number of clinical conditions, in addition to postherpetic neuralgia (10).

However, a few studies have been conducted on additive or synergistic antinociceptive effects of ascorbic acid combined with other analgesics, especially opioid substances. Some studies could show that concurrent use of ascorbic acid decreased morphine consumption in postoperative periods (11). It has been hypothesized that inhibition of morphine tolerance and dependence development by ascorbic acid has two dopaminergic and glutamatergic components (12). However, a detailed evaluation of the combination of ascorbic acid and commonly used analgesics has not been reported.

## 2. Objectives

The purpose of the present study was to assess the nature of the interaction between ascorbic acid and two analgesic drugs, morphine and tramadol, in a rodent model of visceral pain.

## 3. Materials and Methods

Male Naval Medical Research Institute (NMRI) mice (25-30 g), housed on a 12-hour light-dark cycle at 22 ± 2°C with access to food and water, were used in this experimental survey. The experiments were performed in accordance with the Guideline for the Care of Laboratory Animals and the ethical guidelines for investigation of experimental pain, approved by the research Committee of Hamedan University of Medical Sciences. The analgesic activity was assessed by the acetic acid abdominal constriction test (writhing test) (13). Mice were injected intraperitoneally (IP) with 10 mL/kg of 0.7% acetic acid solution, 30 minutes after the IP administration of the study drugs or normal saline as control, the time at which preliminary experiments showed occurrence of the maximum effect. A writhe is characterized by a wave of contraction of the abdominal musculature followed by extension of the hind limbs. The number of writhes in a 10-minute period was counted, starting five minutes after the acetic acid administration. Antinociceptive activity was expressed as the inhibition percentage of the usual number of writhes in a drug-treated animal, when compared to the mean number of writhes obtained in control animals in this study (14).

Dose-response curves for the antinociceptive effects of ascorbic acid, morphine and tramadol were obtained, using at least four to six animals at each of at least four doses. The drugs were injected IP. A least squares linear regression analysis of the log dose-response curves allowed calculation of the dose that caused 50% antinociception (ED_50_) for each drug. Using this protocol, ED_20_ and ED_65_ were also calculated. A dose-response curve was also obtained by IP co-administration of ascorbic acid and morphine as well as ascorbic acid and tramadol in fixed combination ratios of their respective ED_50_, ED_20_ and ED_65_: 1/2, 1/4, 1/8, 1/16 (15). The theoretical additive ED_50_obtained from the calculation, ED_50_ add, was calculated as: ED_50_ ascorbic acid/Pl + RP2, where R is the potency ratio of ascorbic acid alone to the combined drug alone, Pl is the proportion of ascorbic acid and P2 is the proportion of the combined drug in the total mixture (16). The potency ratio in each combination was obtained by ED_50_, ED_20_, or ED65 for morphine or tramadol/ED_50_, ED_20_, or ED_65_ for ascorbic acid, respectively (2).

Supra-additivity or synergistic effect is defined as the effect of a drug combination which is statistically different. If the experimental ED is significantly lower than the theoretically calculated equi-effect of a drug combination with the same proportions, the effect of the combination is synergistic. Otherwise, the effect of the combination is additive and additivity means that each constituent contributes with its own potency to the total effect. The interaction index was calculated as experimental ED/theoretical ED. If the value is close to one, the interaction is additive. Values lower than one are an indication of the magnitude of supra-additive or synergistic interactions and values higher than one correspond to subadditive or antagonistic interactions (17). Furthermore, lower values indicate higher potency of the combinations.

The results were presented as means ± SD or ED50 values with 95% confidence limits (95% CL). The statistical difference between theoretical and experimental values was assessed by student’s t-test for independent means and P values less than 0.05 were considered significant.

## 4. Results

The number of animals was kept at a minimum, compatible with consistent effects of the drug treatments. A few mice had no response to acetic acid and did not show writhe; so, they were omitted and replaced by new ones.

The ED_50_, ED_20_, and ED_65_ values and 95% CL for the antinociceptive effects of the IP studied drugs are shown in Table 1. Our observations showed that the antinociceptive effects of all drugs were dose-dependent (ED_50_was 206.1 mg/kg for ascorbic acid, 8.33 mg/kg for tramadol, and 0.79 mg/kg for morphine). In fact, IP administration of ascorbic acid, morphine, and tramadol showed dose-dependent antinociceptive effects with different potencies in the abdominal constriction test of mice.

The antinociceptive activity of IP co-administration of fixed combination ratios of ED_50_, ED_20_ and ED_65_ of morphine or tramadol and ascorbic acid was assessed by calculating the ED_50_, ED_20_ and ED_65_ of the mixtures from the corresponding dose-response curves.

Table 2 shows the theoretical and experimental ED_50_, ED_20_ and ED_65_ values for the combinations, with their 95% CL and fixed ratios.

Table 3 shows the interaction index of the combination of ascorbic acid and tramadol, administered IP, which resulted in additive interactions at ED_50_ and ED_65_, while led to synergistic interactions at ED_20_. The interaction index of the combination of ascorbic acid and morphine showed additive interactions at ED_50_ and ED_65_; however, synergistic interaction was found at ED_20_, obtained from the dose-response curves. Furthermore, the interaction index values of the IP combinations demonstrated the potency rate of ascorbic acid/morphine to be lower than ascorbic acid/tramadol.

**Table 1.** ED50, ED_20_, and ED_65_ Values with 95% Confidence Limits forAntinociceptive Effects of Ascorbic Acid, Morphine, and Tramadol in the Writhing Test of Mice ^a^

ED	ED, mg/kg (CL)
**ED_50_**	
Ascorbic acid	206.1 (179.1-237.2)
Tramadol	8.33 (6.91-10.09)
Morphine	0.79 (0.58-1.09)
**ED**_**20**_	
Ascorbic acid	82.56 (23.60-288.8)
Tramadol	3.28 (1.75-6.16)
Morphine	0.14 (2.60-1.25)
**ED**_**65**_	
Ascorbic acid	300.50 (218.0-413.2)
Tramadol	24.40 (13.01-45.74)
Morphine	2.70 (2.21-3.29)

^a^ Abbreviations: CL, confidence limit; ED, Effective Dose .

**Table 2.** Theoretical and Experimental ED_50_ ,ED_20_, and ED_65_ Values with 95% Confidence Limits and Ratios for Combinations of Ascorbic Acid with Morphine and Tramadol in the Writhing Test of Mice ^a^

ED and Combination	ED, mg/kg (CL)		Ratio
	Theoretical	Experimental	
**ED_50_**			
TR/AA	107.22 (93.00-123.62)	102.88 (80.86-149.42)	1:24.7
MO/AA	103.44 (89.84-119.14)	105.13 (74.79-144.01)	1:260.89
**ED**_**20**_			
TR/AA	49.92 (12.68-14.48)	29.74 (19.38-45.00)	1:24.7
MO/AA	41.35 (10.50-145.03)	29.12 (23.73-35.73)	1:260.89
**ED**_**65**_			
TR/AA	162.45 (115.76-229.47)	145.05 (115.77-181.74)	1:24.7
MO/AA	151.60 (110.36-208.24)	139.58 (56.09-348.55)	1:260.89

^a^ Abbreviations: AA, ascorbic acid; CL, confidence limit; ED, Effective Dose ; MO, morphine; TR, tramadol.

**Table 3.** Interaction Index of the Combinations of Ascorbic Acid and Morphine or Tramadol in the Writhing Test of Mice^a^

ED	Interaction Index	P Value
**ED_50_**		
AA/TR	0.96	0.27
AA/MO	1.016	0.19
**ED**_**20**_		
AA/TR	0.69	0.009
AA/MO	0.70	0.008
**ED**_**65**_		
AA/TR	0.89	0.81
AA/MO	0.92	0.64

^a^ Abbreviations: AA, ascorbic acid; ED, Effective Dose ; MO, morphine; TR, tramadol.

## 5. Discussion

This study revealed some important findings. First, the antinociceptive effects of three drugs were dose-dependent with different potencies. We wanted to identify the possible antagonism and synergism in presence of high and low levels of antinociception.

Furthermore, using different ED values, combination of ascorbic acid and morphine or tramadol had different interactions, either additive or synergistic, so that the combinations resulted in synergistic interactions at ED_20_, but led to additive interaction at ED_50_ and ED_65_. It has been reported that tramadol and methimazole had antagonism at ED20, whereas at ED_50_ and ED_80_, they showed synergy. The mechanism of interaction has been unknown (18).

Finally and according to the interaction index value, co-administration of ascorbic acid/tramadol had higher potency rate in comparison with that of ascorbic acid and morphine. As shown in a study supplementation with ascorbic acid decreased morphine consumption in the postoperative period, in patients undergoing laparoscopic cholecystectomy (9). It has been demonstrated that local perfusion of morphine can produce increased levels of extracellular ascorbic acid in nervous system of animal models and this morphine-induced changes in ascorbic acid can be mediated by the release of γ-aminobutyric acid (GABA) and dopamine (DA) as well as activation of these hormones specific receptors (19). This effect can also change the motivational processes underlying morphine self-administration. Previous studies have shown that co-administration of morphine and other drugs produced different interactions. Selective imidazoline I_2_ receptor ligands have substantial antinociceptive effects and produce antinociceptive synergy with morphine in a rat model with acute pain (20).

The interaction between morphine and nicotine is additive (21). Morphine and gabapentin have shown antinociceptive synergism in relieving the neuropathic pain induced by chronic constriction injury (22) and ascorbic acid can decrease morphine self-administration and withdrawal symptoms in rats (23). Tramadol has a central analgesic effect and its mechanism is mainly based on serotonin reuptake inhibition (24). It can also inhibit the norepinephrine transporter function (25). The ability of tramadol to create analgesic effect is partly through activating the opioid receptors, indicating that the dose-response curves of these two types of drugs are not parallel.

Combination of tramadol and acetaminophen for painful diabetic neuropathy in streptozotocin-induced diabetic rats produces an additive antinociceptive effect (26). Tramadol and caffeine combination showed synergistic interactions on the antinociception measured in a formalin model (27). Our results showed that the interaction between ascorbic acid and morphine or tramadol on antinociception may be synergistic or additive, depending on the level of effect. Additivity could be demonstrated at 50% or higher levels of the effect.

Different levels of potency ratios can also explain their different pharmacological pathways, which should be more evaluated in further studies. The analysis of antinociceptive efficacies produced by co-administration of ascorbic acid and morphine or tramadol for different levels of nociception, revealed that co-administration provided better antinociceptive coverage than the individual drugs. There is neither a simple explanation for the mechanism of interaction between the two drugs, nor for the type of interaction at different levels of the antinociceptive effect. We had different effects (additive or synergistic) of ascorbic acid in different EDs of analgesics with unknown reasons. We need further researches to determine the mechanism of this interaction. Findings of the present work can be very important, because in clinical settings, combinations of ascorbic acid and tramadol or morphine can decrease the high postoperative dosages of administered opiates, and thus, can lead to less drug side effects.
